# A novel endothelial damage inhibitor for the treatment of vascular conduits in coronary artery bypass grafting: protocol and rationale for the European, multicentre, prospective, observational DuraGraft registry

**DOI:** 10.1186/s13019-019-1010-z

**Published:** 2019-10-15

**Authors:** Etem Caliskan, Sigrid Sandner, Martin Misfeld, Jose Aramendi, Sacha P. Salzberg, Yeong-Hoon Choi, Vilas Satishchandran, Geeta Iyer, Louis P. Perrault, Andreas Böning, Maximilian Y. Emmert

**Affiliations:** 1 Department of Cardiovascular Surgery, Charité Universitätsmedizin Berlin, Berlin, Germany; 2 Department of Cardiothoracic and Vascular Surgery, German Heart Center Berlin, Berlin, Germany; 3 Department of Cardiac Surgery, Vienna General Hospital, Medical University of Vienna, Vienna, Austria; 4 University Clinic of Cardiac Surgery, Heart Center Leipzig, Leipzig, Germany; 5 Division of Cardiac Surgery, Hospital de Cruces, Barakaldo, Spain; 6 Heart Clinic, Hirslanden, Zürich, Switzerland; 7 Department of Cardiothoracic Surgery, Heart Center of the University Hospital of Cologne, Cologne, Germany; 8 Somahlution, Jupiter, FL USA; 9 Department of Surgery, Montreal Heart Institute, Université de Montréal, Montreal, Québec Canada; 10 Department of Cardiovascular Surgery, Justus-Liebig University Gießen, Gießen, Germany

**Keywords:** Saphenous vein graft, Vein graft failure, Myocardial infarction, Coronary artery bypass grafting, Patency, Endothelial damage inhibitor

## Abstract

**Background:**

Vein graft disease (VGD) impairs graft patency rates and long-term outcomes after coronary artery bypass grafting (CABG). DuraGraft is a novel endothelial-damage inhibitor developed to efficiently protect the structural and functional integrity of the vascular endothelium. The DuraGraft registry will evaluate the long-term clinical outcomes of DuraGraft in patients undergoing CABG procedures.

**Methods:**

This ongoing multicentre, prospective observational registry will enrol 3000 patients undergoing an isolated CABG procedure or a combined procedure (ie, CABG plus valve surgery or other surgery) with at least one saphenous vein grafts or one free arterial graft (ie, radial artery or mammary artery). If a patient is enrolled, all free grafts (SVG and arterial will be treated with DuraGraft. Data on baseline, clinical, and angiographic characteristics as well as procedural and clinical events will be collected.

The primary outcome measure is the occurrence of a major adverse cardiac event (MACE; defined as death, non-fatal myocardial-infarction, or need for repeat-revascularisation). Secondary outcome measures are the occurrence of major adverse cardiac and cerebrovascular events (MACCE; defined as death, non-fatal myocardial-infarction, repeat-revascularisation, or stroke), patient-reported quality of life, and health-economic data. Patient assessments will be performed during hospitalisation, at 1-month, 1-year, and annually thereafter to 5 years post-CABG. Events will be adjudicated by an independent clinical events committee. This European, multi-institutional registry will provide detailed insights into clinical outcome associated with DuraGraft.

**Discussion:**

This European, multi-institutional registry will provide detailed insights into clinical outcome associated with the use of DuraGraft. Beyond that, and given the comprehensive data sets comprising of patient, procedural, and graft parameters that are being collected, the registry will enable for multiple subgroup analyses targeting focus groups or specific clinical questions. These may include analysis of subpopulations such as patients with diabetes or multimorbid high-risk patients (patient level), evaluation of relevance of harvesting technique including endoscopic versus open conduit harvesting (procedural level), or particular graft-specific aspects (conduit level).

**Trial registration:**

ClinicalTrials.gov NCT02922088. Registered October 3, 2016.

**Ethics and dissemination:**

The regional ethics committees have approved the registry. Results will be submitted for publication.

## Background

Surgical revascularisation represents the gold standard for patients with multivessel coronary artery disease [1]. However, besides the left internal mammary artery being the established graft for the left anterior descending (LAD) territory, the debate about conduit selection for the other coronary territories is ongoing [2]. Especially for younger patients, multi- or even total arterial grafting strategies are recommended, and recent data indicate a significant long-term benefit when using multiple arterial grafts (ie, right internal mammary artery or radial artery) [3, 4]. To increase the use of multiple arterial grafts, the Society of Thoracic Surgeons recently published the ‘First Clinical Practice Guidelines on Arterial Conduits [5]. Despite the current effort on multiarterial grafting, the adoption rate of multiple arterial conduits in daily clinical practice is rather slow and is still below 10% due to the technical complexity added to the CABG procedure [5]. Instead, saphenous vein grafts (SVGs) remain the most common conduits for coronary artery bypass grafting (CAGB) procedures [5–9]. In most cases, SVG conduits are readily available and easy to harvest, which is particularly beneficial in urgent or emergency cases with ongoing ischaemia. Moreover, SVGs are considered to be more resistant to manipulation during harvest and anastomosis, and appear less susceptible to vasospasm. However, compared with arterial conduits, the long-term patency of SVGs due to vein graft disease (VGD) and consecutive failure remains a major issue, substantially impairing long-term clinical outcomes [10, 11]. One-year failure rates range from 15 to 29%, with at least one graft occluded graft at 12–18 months, and up to 40–50% of failed SVGs at 10 years post CABG [10, 12–14]. Besides general factors such as the overall progress of a patient’s coronary artery disease, the quality of the anastomosis and the target vessel, and postoperative preventive strategies, long-term patency strongly depends on the overall quality of the SVG conduit itself. Importantly, damage to the fragile SVG endothelium appears to be a key promotor for the development and progress of VGD, stenosis, and complete failure. Therefore, to best possibly protect the SVG endothelium, several intraoperative considerations are mandatory. These include state-of-the-art graft harvesting techniques to reduce traumatic injury (ie, no or less-touch techniques), avoidance of graft distension or over-pressurisation (ie, when assessing for potential leakages), and, importantly, effective intraoperative storage and preservation to reduce ischaemia reperfusion injury (IRI) of the graft endothelium between harvest and until completion of anastomosis [10, 11].

To date, (heparinised) saline and autologous whole blood (AWB) are the most frequently used solutions for intraoperative storage and preservation of SVGs [15]. However, multiple experimental studies and a recently published subanalysis of the PREVENT-IV trial have clearly shown that neither of these solutions sufficiently protects the endothelium [14, 16–20], highlighting a strong clinical need for alternative preservation strategies.

DuraGraft (Somahlution Inc., Jupiter, FL) is a one-time intraoperative graft treatment that has been developed to protect against endothelial damage of vascular conduits during CABG. Several in-vitro studies demonstrated superiority for DuraGraft compared with saline or AWB in preserving the functional and structural integrity of the conduits’ endothelium [16, 21]. Furthermore, a retrospective observational study demonstrated that DuraGraft is associated with reduced long-term complications, including the occurrence of myocardial infarction and the need for repeat revascularisation [22]. Despite these encouraging ex-vivo and clinical data, systematic data validating the clinical efficacy of DuraGraft to prevent SVGs from the development of intimal hyperplasia leading to VGD and subsequent VGF are still pending.

The European, multi-institutional DuraGraft registry will assess the potential benefit of DuraGraft to efficiently protect against the development and progression of VGD or VGF in patients undergoing CABG procedures. The objectives of this registry include the long-term assessment of clinical outcomes, including major adverse cardiac events (myocardial infarction, death, repeat revascularisation), quality-of-life data, and health-economic outcomes in patients requiring CABG procedures and whose vascular grafts are treated with DuraGraft.

## Methods/design

### Study design

The DuraGraft registry is a prospective, multicentre, non-randomised observational study involving 38 centres from eight countries in Europe (see Additional file 1: Table S1) for an enrolment target of 3000 patients. A registry advisory committee has been established to oversee the study, and an independent clinical events committee has been set up for adjudication of adverse events which meet protocol defined outcomes/endpoints over the course of the study. The registry is registered at Clinicaltrials.gov under the identifier NCT02922088. Routine baseline, clinical, and angiographic characteristics as well as procedural and clinical events will be recorded using standardised web-based electronic case report forms. A EuroQol questionnaire will collect patient information on quality of life. Hospitalisations and use of healthcare resources after CABG procedures will be used to determine health-economic outcomes. Patient assessments will be performed during hospitalisation, at 1 month, 1 year, and annually thereafter to 5 years post-CABG The study schedule is provided in Table 1.

### Patient population and recruitment

Men or women are eligible to enrol in the study if they meet the following criteria: age ≥ 18 years; undergoing an isolated CABG procedure or a combined procedure (ie, CABG plus valve surgery or others) with at least one (or more) saphenous vein graft(s) or one (or more) free arterial graft(s) (ie, radial artery or internal mammary artery); and DuraGraft is used during the CABG procedure. If a patient is enrolled, all free grafts (venous and/or arterial) will be treated with DuraGraft. Patients are excluded if they are participating in a medical device study or have received an investigational study drug in the month before enrolment.

Consecutive eligible patients are being enrolled at all 38 participating centres for a total of 3000 patients. The first patient was enrolled on 9 December 2016. The registry is currently active at 38 sites throughout Europe and 2700 patients had been enrolled by June 2019. Completion of enrolment is anticipated in Q3 2019 with the short term data (30 days) to become available in Q1/2020, followed by 1-year and 5-year follow up data in 2021 and 2025 respectively.

### Intervention

DuraGraft (Somahlution, Jupiter, Florida, USA) is an intraoperative treatment that protects against damage to the structure and function of the vascular endothelium [22]. It comes in two containers and is formulated into an ionically and pH-balanced physiological salt solution containing glutathione, L-ascorbic acid, L-arginine, and other ingredients that protect the conduit from the damaging effects of ischaemia that occurs during storage and reperfusion injury during CABG. In brief, after harvest, every SVG or the free arterial graft (i.e. radial artery, internal mammary artery) will be carefully flushed with and stored within DuraGraft prior to anastomosis [23]. Grafting strategy and configuration is left to the discretion of the surgeon.

### Data collection and quality assurance

Electronic case report forms will be completed by trained study coordinator(s) at each site. The following data will be captured: patient demographics; baseline clinical and angiographic characteristics; medical history and EuroSCORE II [24]; cardiac operative and postoperative data; graft and anastomosis characteristics (i.e. grafting strategy and configuration); haemodynamic variables; cardiovascular medications; patient-reported quality-of-life data; and health-economics data. A summary of collected parameters can be found in Additional file 1: Table S2.

To maintain confidentiality, a unique identification number will be allocated to each patient enrolled in the registry. Anonymised patient-level data will be submitted to a centralised database (Dendrite Clinical Systems, Henley-on-Thames, Oxfordshire, UK).

During the follow-up period, patients will be contacted via mail, email or telephone at 1 month, 1 year, and annually thereafter to 5 years following the CABG procedure to determine whether cardiac-related adverse events and/or hospitalisations have occurred since the last follow up. For patients who report such hospitalisations, the study coordinator at the participating site will contact the hospital/practice where the patient was hospitalised to request additional information to be entered into the registry database.

All electronic case report forms will be reviewed and checked for entry errors, and data queries requiring clarification will be sent to the study site for resolution. Source data verification will be performed on a random sample of patients selected from across all study sites.

### Outcome measures

The primary outcome measure is the occurrence of a major adverse cardiac event (MACE; defined as death, non-fatal myocardial infarction, or need for repeat revascularisation) at 1 month, 1 year, and annually thereafter to 5 years post-CABG. Secondary outcome measures are the occurrence of major adverse cardiac and cerebrovascular events (MACCE; defined as death, non-fatal myocardial infarction, repeat revascularisation, or stroke), patient-reported quality of life, and health-economics data. Definitions for outcome measures are provided in Table 2.

**Table 1:** Study schedule

	Screening/ Enrolment	CABG/ hospital discharge	1 month	Annual follow-up (1–5 years)
Inclusion and exclusion criteria	X			
Informed consent	X			
Patient characteristics/medical history	X			
EuroSCORE II	X			
Procedural data		X		
EQ-5D-5 L	X			X
Health economic data derived			X	X
MACCE		X	X	X

*MACCE* Major adverse cardiac and cerebrovascular events (death, non-fatal myocardial infarction, repeat revascularisation, or stroke)

**Table 2:** Definitions of registry outcome measures

Outcome	Definition
Mortality	
Cardiovascular	Any of the following criteria: • Death due to proximate cardiac cause (eg, myocardial infarction, cardiac tamponade, worsening heart failure) • Death caused by non-coronary vascular conditions such as neurological events, pulmonary embolism, ruptured aortic aneurysm, dissecting aneurysm, or other vascular disease • All procedure-related deaths, including those related to a complication of the procedure or treatment for a complication of the procedure • All valve-related deaths including structural or non-structural valve dysfunction or other valve-related adverse events • Sudden or unwitnessed death • Arrhythmia or cardiac arrest • Death of unknown cause
Non-cardiovascular	Any death in which the primary cause of death is clearly related to another condition (e.g. trauma, cancer, suicide)
Myocardial infarction	
Periprocedural	≤48 h after the index procedure: absolute rise in cardiac troponin (from baseline) ≥35 times upper reference limit plus ≥1 of the following criteria: • New significant Q waves or equivalent • Flow-limiting angiographic complications • New “substantial” loss of myocardium on imaging
Spontaneous	> 48 h after the index procedure: detection of rise and/or fall in cardiac biomarkers with ≥1 value above the 99th percentile upper reference limit, together with the evidence of myocardial ischaemia with ≥1 of the following present: • Symptoms of ischaemia • Electrocardiographic changes indicative of new ischaemia (new ST-T changes or new left bundle branch block • New pathological Q-waves in ≥2 contiguous leads • Imaging evidence of a new loss of viable myocardium or new wall motion abnormality • Sudden, unexpected cardiac death, involving cardiac arrest, often with symptoms suggestive of myocardial ischaemia, and accompanied by presumably new ST elevation, or new left bundle branch block, and/or evidence of fresh thrombus by coronary angiography and/or at autopsy, but death occurring before blood samples could be obtained, or at a time before the appearance of cardiac biomarkers in the blood • Pathological findings of an acute myocardial infarction
Revascularisation	Endovascular or surgical procedure performed on the DuraGraft-treated venous or arterial graft(s) because of loss of graft patency
Stroke	Neurological deficit documented by physical examination or some form of imaging

For the quality-of-life data, patients will be required to complete the EQ-5D-5 L [25] health status instrument at baseline (before the CABG surgery), at 1 year, and annually thereafter to 5 years. EQ-5D-5 L comprises five questions, each with five levels that represent five health domains: pain, mood, mobility, self-care, and activities of daily living [25]. The registry data will be presented as a measure of overall self-rated health status.

### Statistical design and analyses

The data will be presented using descriptive statistics, including estimates of event rates, confidence intervals, and basic cross-tabulations of all data elements.

### Registry status

Enrolment is ongoing and is expected to be completed by Q3/2019 with first data expected to become available by Q4/2019.

## Discussion and expected outcomes

VGD and subsequent failure still represent a major issue in CABG surgery [10, 11]. Structural and functional damage of the conduit’s endothelium due to mechanical harm during harvest and handling, and, importantly, to ineffective intraoperative preservation appear to be one of the main triggers of VGD which can be divided into three mechanisms that may occur at different phases post-grafting: (a) thrombosis (early phase, within hours to < 1 month); (b) intimal hyperplasia (intermediate phase, within months); and (c) atherosclerosis (late phase, > 12 months) [10].

Duragraft is isotonic, pH-neutral and -buffered and contains potent antioxidants and a substrate for endothelial nitric oxide synthase. This biocompatible milieu with endothelial protective, as well as ischaemic reperfusion, ischaemia-preventive properties maintains cell endothelial cell viability and integrity, thereby inhibiting the perpetual cascade of maladaptive processes that start during graft harvesting and storage. This is in comparison to widely used storage solutions such as heparinised saline (pH 5.5), whole autologous blood (pH 8.0) or pH-buffered solutions that merely prevent changes in pH but do not offer further functionality in maintaining cell integrity.

In this European, multi-institutional registry, the long-term effect of DuraGraft in patients undergoing CABG procedures will be investigated. Both early and long-term data on clinical outcomes, comprising MACE as well as quality-of-life and health-economic data, will be prospectively collected, providing detailed insights into clinical outcome associated with the use of DuraGraft.

The first clinical outcomes from a large, retrospective observational study in patients undergoing isolated CABG demonstrated that SVG treatment with DuraGraft was associated with a reduced rate of long-term adverse events, including myocardial infarction (hazard ratio [HR] 0.55; 95% confidence interval [CI] 0.41–0.74; *p* < 0.0001) and the need for repeat revascularisation (HR 0.65; 95% CI 0.44–0.97; *p* = 0.037), suggesting that intraoperative treatment with DuraGraft may reduce VGD-related adverse events [22]. Taking these first encouraging data into account, the DuraGraft registry aims to validate these findings in a prospective manner, and will also further expand the overall clinical experience with DuraGraft in routine CABG procedures. Beyond that, and given the comprehensive data sets comprising of patient, procedural, and graft parameters that are being collected, the registry will also provide a valuable opportunity for multiple subgroup analyses targeting focus groups or specific clinical questions. These may include analysis of subpopulations such as patients with diabetes or multi-morbid high-risk patients (patient level), evaluation of relevance of harvesting technique including endoscopic versus open conduit harvesting (procedural level), or particular graft-specific aspects (conduit level). Importantly, the registry will also collect the first clinical data on free arterial grafts (ie, right internal mammary artery or radial artery) that have undergone treatment with DuraGraft. In parallel to this registry, a prospective, randomised trial is underway in patients undergoing isolated CABG with at least two SVGs, which will specifically evaluate the graft remodelling behaviour after DuraGraft treatment (NCT02272582 and NCT02774824). DuraGraft-treated SVGs were randomised against saline-treated conduits in the same patient, who were then followed up using sequential multidetector computed tomography angiography [23]; the results are eagerly awaited.

In general, when implementing and validating new devices or treatments, prospectively collected data from large clinical registries have been established as a useful component in creating the body of evidence as they substantially differ from randomised controlled trials (RCTs) in many aspects.

RCTs generally focus on stringently defined patient cohorts with very narrow inclusion and exclusion criteria and usually focus on pre-specified time windows rather than a “real-world” setting. However, as a complement to RCTs with inherent limitations, large-scale observational clinical registries offer a great opportunity to collect data on larger and more diverse patient cohorts that are representative of patients treated in everyday clinical practice. In addition, registries offer follow-up periods that usually extend beyond the classical RCT windows. Moreover, the relevance of findings can be further increased when they project data from different geographical regions, including clinical sites of various sizes with different referral, pre- and perioperative strategies in place [26]. Therefore, comprising numerous small-, mid-, and high-volume European sites, the DuraGraft registry is expected to deliver valuable “real-world” data and insights into current CABG practices from across Europe. Moreover, the fact that all-comers, either undergoing isolated CABG or combined procedures (e.g. valve plus CABG), can be enrolled will further enhance its clinical relevance. Given its representative nature, the registry may also serve as a substantial resource for further comparison against other ongoing or future studies in the field of CABG.

Finally, it must be emphasised that despite extensive evidence of their deleterious effects to the conduits’ endothelium, saline and AWB still represent the most frequently used solutions for intraoperative graft preservation [11, 14, 16–20]. This was highlighted recently in a study comprising data from 100 top-performing US hospitals, with usage in > 55% of patients [15]. Therefore, from an educational perspective, the registry may create further awareness that intraoperative graft preservation represents an important issue and that there is still an urgent need for improved graft preservation strategies, which will be mandatory to achieve the ultimate goal of enhanced SVG patency, thereby reducing long-term complications after CABG.

## Supplementary information

**Additional file 1: Table S1.** List of institutions and principal investigators. **Table S2.** List of preoperative parameters that are collected in the case report forms.

## Data Availability

Not applicable.
