# Supplementary material for: A novel endothelial damage inhibitor for the treatment of vascular conduits in coronary artery bypass grafting: protocol and rationale for the European, multicentre, prospective, observational DuraGraft registry
Source: J Cardiothorac Surg. 2019 Oct 15;14:174. doi: 10.1186/s13019-019-1010-z (PMC6794868; doi:10.1186/s13019-019-1010-z)
Supplement: Supplementary file 1 — Additional file 1: Table S1. List of institutions and principal investigators. Table S2. List of preoperative parameters that are collected in the case report forms. [file 13019_2019_1010_MOESM1_ESM.docx]

**Additional file 1**

**Table S1** List of institutions and principal investigators

| **Country** | **City, Institution** | **Principal investigator** |
| --- | --- | --- |
| Austria | Vienna, Medical University of Vienna | Professor Sigrid Sandner |
| Austria | Innsbruck, Innsbruck Medical University | Dr Cenk özpeker |
| Germany | Berlin, Charité University of Medicine Berlin | Dr Ibrahim Caliskan |
| Germany | Berlin, German Heart Center Berlin | Dr Jörg Kempfert |
| Germany | Dresden, Herzzentrum Dresden | Professor Habil Klaus Matschke |
| Germany | Göttingen, Georg August University of Göttingen | Dr Bernd Danner |
| Germany | Aachen, RWTH Aachen University | Professor Rüdiger Autschbach |
| Germany | Cologne, Cologne University Heart Center | Professor Yeong Hoon Choi |
| Germany | Giessen, University of Giessen | Professor Andreas Böning |
| Germany | Leipzig, Helios Herzzentrum | Professor Martin Misfeld |
| Germany | Muenchen, German Heart Center Muenchen | Dr Johannes Böhm |
| Germany | Essen, University Hospital Essen | Professor Matthias Thielmann |
| Germany | Frankfurt, Goethe University Hospital Frankfurt | Dr Tomas Holubec |
| Germany | Lübeck, University of Lübeck | Dr Anas Aboud |
| Germany | Wuppertal, Helios Clinic Wuppertal Heart Center | Professor Herbert Vetter |
| Germany | Kiel, University Kiel | Dr Assad Haneya |
| Ireland | Galway, Galway University Hospitals | Dr Dave Veerasingam |
| Ireland | Cork, Cork University Hospital | Dr Kishore Doddakula |
| Italy | Roma, European Hospital | Dr Luca Weltert |
| Italy | Catanzaro, Università Magna Graecia di Catanzaro | Professor Pasquale Mastroroberto |
| Italy | Vicenza, Ospedale Cicile "San Bortolo" | Dr Nicola Lamascese |
| Spain | Badajoz, Complejo Hospitalario Universitario de Badajoz | Dr José Ramón Gonzalez |
| Spain | Madrid, Hospital Universitario Gregorio Maran᷉on | Dr Gregorio Cuerpo Caballero |
| Spain | Madrid, Hospital Ramón y Cajal | Dr Jose Lopez |
| Spain | Salamanca, Salamanca University Hospital | Professor José González-Santos |
| Spain | Seville, Hospital Universitario Virgen Macarena | Dr Juan Carlos Tellez |
| Spain | Santiago de Compostela, Hospital Universitario | Professor Angel Fernandez |
| Spain | Cadiz, Hospital Universitario Puerto del Mar | Dr Tomas Daroca Martinez |
| Spain | Barakaldo, Cruces University Hospital | Dr Jose Aramendi |
| Spain | Cordoba, Reina Sofia University Hospital | Dr Ignacio Munoz |
| Spain | Seville, H.U. Virgen del Rocío | Dr José Borrego Domínguez |
| Spain | Madrid, Ruber International Hospital | Dr Paloma Martinez |
| Switzerland | Lugano, Cardiocentro Ticino | Professor Enrico Ferrari |
| Switzerland | Zurich, University Hospital of Zurich | Dr André Plass |
| Switzerland | Zurich, Herzklinik Hirslanden | Dr Sacha Salzberg |
| Turkey | Antalya, Medical Park Hospital | Dr Ilker Tekin |
| UK | Blackpool, Victoria Blackpool Hospital | Mr Amal K Bose |
| UK | Clydebank, Glasgow, Golden Jubilee National Hospital | Professor Nawar Al Attar |

**Table S2** List of preoperative parameters that are collected in the case report forms

| **Patient characteristics at baseline** |
| --- |
| - Age |
| - Sex |
| - Height |
| - Weight |
| - Smoking history (ex-smoker, currently smoking) |
| - Diabetes mellitus (insulin dependent, non-insulin dependent) |
| - Hyperlipidaemia |
| - Hypertension |
| - Cerebrovascular disease |
| - Peripheral vascular disease |
| - Respiratory disease |
| - Renal disease |
| - Previous myocardial infarction |
| - Previous percutaneous coronary intervention |
| - Previous cardiac surgery (bypass, valve, congenital) |
| - CCS angina class |
| - NYHA functional class |
| - EuroSCORE II |
| - Left main coronary artery disease |
| - Number of diseased coronary arteries |
| - Left ventricular ejection fraction |

CCS, Canadian Cardiovascular Society; NYHA, New York Heart Association.
